# Pneumococcal Vaccination Uptake in People With Immunosuppressed Conditions Using Real-World Primary Care Data Across England: Protocol for a Retrospective Descriptive Study

**DOI:** 10.2196/92266

**Published:** 2026-07-09

**Authors:** Bernardo Meza, Gavin Jamie, Jose Ordóñez-Mena, Rachel Byford, Timea Suli, Michael Bride, Benoît Corriveau, Michael Feher, Martin Whyte, Mark Joy, Simon de Lusignan

**Affiliations:** 1 Nuffield Department of Primary Care Health Sciences, University of Oxford, Radcliffe Primary Care Building, Radcliffe Observatory Quarter, Woodstock Road, Oxford, Oxfordshire, OX2 6GG, United Kingdom, 44 01865 617855; 2 School of Biosciences and Medicine, University of Surrey, Guildford, England, United Kingdom

**Keywords:** pneumococcal vaccination, routine care data, electronic health records, immunosuppression, vaccine uptake, primary care, real-world evidence, retrospective cohort study

## Abstract

**Background:**

The introduction of pneumococcal vaccination programs in the United Kingdom has led to substantial reduction in the burden of pneumococcal disease in the general population, decreasing the incidence of invasive pneumococcal disease and preventing associated mortality. Current UK guidelines recommend pneumococcal vaccination for adults aged ≥65 years, as well as for individuals aged ≥2 years with underlying medical conditions that place them at increased risk of severe pneumococcal disease. This includes adults with immunosuppression. To date, there are few data in the United Kingdom of pneumococcal vaccine coverage in specific high-risk groups, such as those with immunocompromised conditions.

**Objective:**

We aim to evaluate the yearly uptake of pneumococcal vaccine in adults who are included in national recommendations as people with immunosuppressive conditions, stratified by etiology of immunosuppression.

**Methods:**

This will be a retrospective cohort study with data from the Oxford-Royal College of General Practitioners Research and Surveillance Centre network, which is nationally representative of the English population. The population under study includes adults registered in the Research and Surveillance Centre database with immunosuppression, including those with bone marrow compromise, with solid organ transplant, receiving oncological treatment, using immunosuppressive drugs, or with primary or acquired immunodeficiencies. The exposure is the underlying medical condition leading to an immunosuppression category. The primary outcome will be pneumococcal vaccination, defined as one dose of the 23-valent pneumococcal polysaccharide vaccine (PPV23).

**Results:**

The study was funded in May 2025, and data extraction was performed from December 2025 to February 2026. Analysis was started in March 2026, with final results expected to be submited for publication in 2026. We will report pneumococcal vaccine uptake disaggregated for the high-risk group of people with immunosuppressive conditions, which has not been previously reported. We will also report on the socioeconomic gradient for vaccine uptake (through the use of the Index of Multiple Deprivation score and region) and report on the differences among ethnic groups.

**Conclusions:**

We will inform on the granularity of routine primary care data to include disaggregated reports of vaccine uptake in the immunosuppressed population in routine surveillance in the United Kingdom. This will aim to address the gap on pneumococcal vaccination coverage in people with immunosuppressive conditions, helping to identify potential unwarranted variations in vaccine adoption.

## Introduction

### Background

Pneumococcal disease is caused by *Streptococcus pneumoniae*, a bacteria transmitted by aerosols and droplets from respiratory secretions. Several serotypes are associated with mild upper respiratory tract infection and noninvasive disease (eg, otitis media, sinusitis, and bronchitis); however, *S pneumoniae* may also be associated with invasive pneumococcal disease (IPD). IPD is a consequence of infection spreading to a sterile compartment such as blood, cerebrospinal, pleural, joint, or pericardial fluid and is a major cause of morbidity and mortality across all age groups [1,2].

In 1990, only the pneumococcal polysaccharide vaccine (PPV) was available in the United Kingdom. The first pneumococcal conjugate vaccine (PCV) to be introduced in the United Kingdom was PCV7, in 2006, for children. This was replaced in 2013 by PCV13, indicated for children and adults. PPV23 (23-valent pneumococcal polysaccharide vaccine) was available from 1992 as a single dose for clinical risk groups aged ≥2 years, and since 2003, for adults aged ≥65 years [2,3].

The introduction of pneumococcal vaccine programs can be associated with a significant reduction in pneumococcal disease across the general population [2]. One study estimated that, over a 10-year period, a PCV program prevented approximately 38,400 cases of clinical IPD and averted 2000 deaths in the United Kingdom, with similar findings observed in other countries [4,5]. However, since the time of the COVID-19 pandemic, there has been a slight increase in IPD incidence or no evidence of a continuation of the prepandemic progressive reductions [6,7]. The current recommendations for adult pneumococcal vaccination in the United Kingdom include adults aged ≥65 years of age or those aged 2 years and older but with other medical conditions placing them at higher risk of serious pneumococcal disease. These risks groups include individuals with immunocompromising conditions; chronic respiratory, kidney, liver, or heart disease; diabetes; and those with occupational exposure risks [2].

Various definitions of immunosuppression exist, generally based on different combinations of underlying immunosuppressive conditions, medication use, or treatments of specific duration. In the United Kingdom, immunosuppression is defined differently for populations eligible for pneumococcus, flu, or COVID-19 vaccines [8,9]. However, immunosuppression prevalence is generally considered to be around 3%, although more recent US survey data suggests that it is as high as 6.6% of the general population [10]. A large population-based cohort analysis from England during the COVID-19 pandemic estimated that over 500,000 individuals were classified as immunocompromised, primarily owing to immunosuppressive drug therapy or organ transplantation [11].

Stratifying pneumococcal vaccine uptake by etiology of immunosuppression is important due to the diverse mechanisms and severity of immune dysfunction across different conditions. For example, HIV infection primarily affects T cell function, while chemotherapy and hematological malignancies cause broader immune suppression, and immunosuppressive drugs may target specific immune pathways. These variations influence both disease susceptibility and vaccine responsiveness, necessitating tailored vaccination strategies. Stratification also enables targeted public health interventions by identifying subgroups with low uptake.

To date, there are limited data in the United Kingdom on pneumococcal vaccine coverage in specific high-risk groups, such as those with immunocompromised conditions (eg, transplantation recipients, treated or untreated hematological or solid organ cancers). The UK Health Security Agency provides accurate data on pneumococcal vaccination among at-risk populations, including those with asplenia, chronic heart disease, chronic liver disease, chronic kidney disease, chronic respiratory disease, diabetes, and immunosuppression [12]. However, for the immunosuppressed population specifically, available data are aggregated across all causes of immunosuppression. This prevents differentiation in vaccine uptake between distinct etiologies, such as sickle cell anemia, immunosuppressive drug use, primary immunodeficiencies, or HIV infection. Individuals with immunosuppressive conditions have reportedly higher pneumococcal vaccination rates than other risk groups [13]. Therefore, further stratification by immunosuppressive etiology is necessary to identify differences among people with specific underlying causes of their disease.

It is also expected that different etiologies of immunosuppression are associated with distinct sociodemographic profiles, as well as varying levels of accessibility to and engagement with primary health care services. For example, in the United Kingdom, 39% of immunosuppressed people received pneumococcal vaccination between 2022 and 2024 [12]. However, certain subgroups may have lower uptake, which may be due to a lack of clarity or consistency in clinical guidelines. For example, in patients with myeloma, the guidelines state it is not possible to provide clear recommendations regarding the optimal timing of vaccination. This ambiguity may affect uptake, as health care providers are left uncertain about when to vaccinate their patients.

Measuring the background characteristics of high-risk patients is essential for identifying potential sociodemographic and ethnic disparities that influence vaccination uptake and ultimately affect clinical outcomes. As guidelines allow for some clinical discretion, variation between practices could also inform on vaccine uptake among immunosuppressed individuals.

### Aims and Objectives

We aim to evaluate the annual uptake of the pneumococcal vaccine (a single vaccine dose of PPV23) among adults identified in national guidelines as having immunosuppressive conditions, stratified by the underlying etiology of immunosuppression.

The study objectives are (1) to identify a cohort of people who are eligible for pneumococcal vaccination and have an immunosuppressive condition, recognized as a high-risk group as according to guidelines, and (2) to report the yearly frequency of uptake of pneumococcal vaccine in the study cohort, stratified by immunosuppression etiology, sociodemographic characteristics, history of comorbidities, and general practice characteristics.

## Methods

### Data Source

We will use the primary care data from the Oxford-Royal College of General Practitioners Clinical Informatics Digital Hub (ORCHID) trusted research environment [14].

ORCHID data are sourced from the Oxford-Royal College of General Practitioners (RCGP) Research and Surveillance Centre (RSC) [15]. The RSC is one of Europe’s oldest sentinel systems [16], which comprises computerized medical record (CMR) data from general practices distributed across England. The data included in the RSC network and which can be extracted for analysis include demographics, coded diagnostic, laboratory test, and prescription and vaccination data, and it is representative of patients attending primary care across urban and nonurban practices.

Pseudonymized data for this study will be extracted from the RSC network, which includes 1704 general practices in England, recruited to be a nationally representative sample of 19 million registered patients [17,18]. The RSC has over 55 years of experience of infectious disease surveillance including vaccination coverage and effectiveness studies [18-20]. A previous study using RSC data included 421,962 individuals with records of immunosuppressive conditions which could potentially be eligible for inclusion in this study [21].

### Study Design

This will be a retrospective observational study using CMRs from general practices in England contributing to the RCGP RSC network for the period between January 1, 2014, and December 31, 2024.

We will estimate pneumococcal vaccination among immunosuppressed patients and investigate sociodemographic variables measured as a proportion of the eligible population. We will also examine panel data of incidence rates by year.

### Study Population

The study population comprises adults registered in the RSC database who are eligible for pneumococcal vaccination due to their immunosuppressive status, based on the following inclusion criteria:

- Adults aged 18 years and above.
- Registered in the RSC database during the period between January 1, 2014, and December 31, 2024.
- Belonging to the high-risk group of patients with immunosuppression, including the following categories:
  - Bone marrow compromise: disaggregating for bone marrow transplant and malignancies (leukemia and Hodgkin and non-Hodgkin lymphomas).
  - Solid organ transplant
  - Cancer treatment
  - Immunosuppressive drug use (including anticancer drugs)
  - Immunodeficiency: Disaggregating for HIV infection, acquired immunodeficiency, and primary immunodeficiency.
  - Other vulnerabilities including asplenia, celiac disease, sickle cell disease, cochlear implant, and cerebrospinal fluid leak.

The following exclusion criteria will be applied:

- Missing key sociodemographic data. We will exclude people whose age or sex are not recorded.
- Record of contraindication to the pneumococcal vaccine or any of its components.
- Records with data anomalies that impede analysis, such as death records preceding a vaccination record.

### Variables

Variables will be curated by identifying codes related to specific conditions within CMRs using Systematized Nomenclature of Medicine Clinical Terms (SNOMED CT), in accordance with NHS England guidance [22]. Baseline covariates will be reported using the earliest date of the immunosuppressive condition diagnosis as baseline.

The exposure will be the underlying immunosuppressive condition considered as being in the high-risk group by current UK guidelines [2]. The immunosuppression group comprises bone marrow compromise, solid organ transplant, cancer treatment, immunosuppressive drug use, immunodeficiency, and other vulnerabilities. We will develop a clinically informed hierarchical ontology to classify individuals with multiple immunosuppressive conditions recorded on the same index date, assigning a single condition when several diagnoses share the earliest recorded date of immunosuppression.

The primary outcome will be pneumococcal vaccination, as one vaccine dose of PPV23. Comparisons will be made among vaccinated and unvaccinated people, and across specific etiologies for patients with immunosuppressive conditions.

Sociodemographic covariates will be reported at baseline. These include age, sex, ethnicity, demography (urban or rural living), region, general practice–level characteristics, socioeconomic status (measured using the Index of Multiple Deprivation), obesity, alcohol consumption, and smoking status. History of comorbid conditions will be extracted from 10 years of CMRs, including chronic kidney disease, type 1 and type 2 diabetes, and cardiometabolic disease. The Cambridge Multimorbidity Score will be used to report on comorbidity profiles [23,24].

General practice characteristics will include geographical distribution (NHS Region), demography (urban/rural), practice size (eg, number of physicians, nurses, and patients), patient turnover indicators, and performance indicators, as available.

### Statistical Analysis

We will estimate yearly vaccination rates of pneumococcal vaccination among people with a record of immunosuppressive conditions. Pneumococcal vaccination rates will be calculated using the number of vaccinated people in high-risk groups as the numerator and estimates of the total high-risk population in the RSC dataset as the denominator. In addition, we will examine panel data of incidence rates by year.

We will describe covariates among the study population, measured as a proportion of the eligible population, including immunosuppressive etiology, sociodemographic characteristics, and comorbidities. Baseline characteristics of each study group will be summarized using descriptive statistics including measures of dispersion (eg, standard deviation and interquartile ranges).

To compare individual characteristics by vaccination status, we will use descriptive statistics, with pairwise comparisons using standardized mean differences, chi-square tests for categorical variables, and *t* or Wilcoxon tests for continuous variables. All statistical tests will be 2-sided, with *P*<.05 considered statistically significant.

Descriptive analyses will be conducted on the overall study population and applicable subgroups. Data that are not documented in our database will be reported as missing. Missing data will be presented as a separate category.

Sensitivity analyses will explore the impact of the hierarchical ontology by classifying individuals with multiple immunosuppressive conditions recorded on the same index date as a separate category.

All analyses will be carried out in the R programming language [25].

### Ethical Considerations

Anonymized electronic health record data were accessible for research purposes following ethical approval from the Central University Research Ethics Committee at the University of Oxford (MSIDREC-1622132) for human participant research. Anonymized patient data were retrieved from electronic health record providers from those users who had not dissented for their data to be used for secondary research purposes. No form of compensation was provided to participants.

We aim to make the study protocol publicly available in accordance with Open Science Framework recommendations to promote transparency, facilitate evaluation of findings against prespecified objectives, and minimize duplication of research efforts.

## Results

This study will present a use case for monitoring the implementation of preventive care interventions using real-world data from a representative sample of the adult population in England.

This study will report disaggregated data for the high-risk group of people with immunosuppressive conditions, which have not been previously published. It will include analysis of the socioeconomic gradient in vaccine uptake—assessed using the Index of Multiple Deprivation score and geographical region—as well as differences in uptake across ethnic groups and across general practices. The study will also examine pneumococcal vaccine uptake during the COVID-19 pandemic period, which can inform on the potential disparities in access to routine preventive measures in future pandemics.

The study will present an ontology for immunosuppressive conditions, contributing to CMR research following open science frameworks for reproducible research.

The study was funded in May 2025, and data extraction was performed from December 2025 to February 2026. Analysis was started in March 2026, with final results expected to be submited for publication in 2026. We aim to publish the findings together with the relevant supporting documentation, including the ontology and associated SNOMED CT code lists.

## Discussion

### Principal Findings

This study aims to demonstrate to health policy decision-makers the importance of incorporating a disaggregated analysis of individuals with immunosuppressive conditions into the routine monitoring of pneumococcal vaccination uptake conducted by the UK health authorities. Such an approach is intended to strengthen the prevention of pneumococcal disease in this vulnerable population and support adherence to official vaccination recommendations. We will assess whether routinely collected health records possess sufficient data quality and granularity to effectively perform a disaggregated analysis of pneumococcal vaccine uptake among immunosuppressed individuals [12]. Furthermore, the proposed ontological framework would also allow for granular data of clinical interest to be accessible within the existing routine dataset resources.

This study will address the data gap in pneumococcal vaccination coverage in people with immunosuppressive conditions. Relying solely on aggregated data for immunosuppressed individuals may obscure vaccination coverage across various demographic and clinical subgroups. For example, a study of individuals with systemic lupus erythematosus in the United States found that those treated with low-intensity immunosuppression were significantly less likely to receive a pneumococcal vaccine recommendation compared to those receiving more intense immunosuppressive therapy [26]. Similarly, vaccination was also more likely in older individuals and those receiving disease-modifying antirheumatic drugs as compared to those only receiving steroids [26]. In contrast, a study conducted on the general population in Belgium reported that pneumococcal vaccination was more likely in those with higher risk conditions. However, disaggregation of vaccine uptake by specific high-risk etiologies has not yet been reported [27].

We aim to report on potential unwarranted variations in pneumococcal vaccine adoption across regional and sociodemographic disparities. Higher odds of vaccination have been reported in urban areas and among people with higher literacy [28]. International studies in the United States and Belgium reported that pneumococcal vaccination is more likely in people with a previous influenza vaccine and less likely in people with low socioeconomic status [27,28]. Similarly, in the United States, differences have been reported between Black and non-Black populations in the likelihood of having conditions conferring a high risk of pneumococcal disease, which can impact the length of stay and costs of pneumococcal disease hospitalizations [29]. As guidelines allow for some clinical discretion, investigating variation between practices can also identify targets for developing practice-level interventions to improve uptake [30]. Overall, there exists scope for identifying target subgroups in the immunosuppressed population for whom interventions for improving vaccine uptake could be implemented, such as nudges for opportunistic vaccination during routine primary care visits or through dual vaccination during the administration of other commonly used vaccines, such as the seasonal flu vaccine.

Lastly, this study will contribute to the development and creation of ontologies and phenotypes for identifying individuals with specific conditions in electronic health records using SNOMED CT, in line with NHS England standards [22]. This code-based phenotyping approach enhances reproducibility in epidemiological studies and facilitates more comprehensive reporting when using routine datasets. Ontologies offer a reproducible method for the hierarchical stratification of overlapping diagnoses across disease categories, such as renal disease versus immunosuppressive drug treatment. This approach aligns with the goals of transparent and reproducible research using CMR data.

### Strengths and Limitations

Our study will not include linkage to secondary care data, hence not capturing the records of therapies prescribed in secondary care. However, this is mitigated by the appropriate recording in the primary care record of long-term immunosuppressive therapies that were initially prescribed in secondary care, which are recorded for the follow-up of adverse events (eg, methotrexate).

There exist various definitions of immunosuppression across flu, COVID-19, and pneumococcus vaccination guidelines in the United Kingdom. However, we investigated the specific definition for pneumococcal vaccination pertinent to the intervention under study. We also developed an ontology to capture diagnoses of immunosuppression under varying criteria, actionable for further research.

### Conclusion

We will evaluate the availability and granularity of routine primary care data to inform the development of disaggregated vaccine uptake reporting for immunosuppressed populations within UK surveillance programs.

This will aim to address the gap in pneumococcal vaccination coverage in people with immunosuppressive conditions. This may help to identify potential unwarranted variations in vaccine adoption, for example, across sociodemographic determinants, disease etiology, or immunosuppressive therapy received.

Immunosuppressed patients are at a high risk of developing invasive pneumococcal disease. Information on vaccination coverage in the United Kingdom should routinely identify factors affecting pneumococcal vaccine uptake to enable the design of public health interventions that improve vaccine uptake.

## Abbreviations

CMR: computerized medical record
IPD: invasive pneumococcal disease
ORCHID: Oxford-Royal College of General Practitioners Clinical Informatics Digital Hub
PCV: pneumococcal conjugate vaccine
PPV: pneumococcal polysaccharide vaccine
PPV23: 23-valent pneumococcal polysaccharide vaccine
RCGP: Royal College of General Practitioners
RSC: Research and Surveillance Centre
SNOMED CT: Systematized Nomenclature of Medicine Clinical Terms
